# Correlation Analysis of Ocular Symptoms and Signs in Patients with Dry Eye

**DOI:** 10.1155/2017/1247138

**Published:** 2017-02-20

**Authors:** Hang Song, Mingzhou Zhang, Xiaodan Hu, Kaixiu Li, Xiaodan Jiang, Yan Liu, Huibin Lv, Xuemin Li

**Affiliations:** Department of Ophthalmology, Peking University Third Hospital, Beijing, China

## Abstract

*Purpose*. To analyze the correlations between the ocular surface signs and symptoms in patients with dry eye.* Methods.* In this observational study, 176 dry eye patients, including 60 males and 116 females, were enrolled and their dry eye symptoms and ocular signs were observed. Partial correlation analysis was conducted between OSDI score and each ocular surface sign, and the correlations were further discussed in different age groups. Then multiple linear regression analysis was used to further explore the influence of these signs on OSDI score.* Results*. Our correlation analyses showed that rounding of lid margins, notching of lid margins, vascularity of lid margins, hyperkeratinization, plugging of orifices, main duct dropout, and conjunctival congestion all had a positive correlation with OSDI score, while main duct number (central 1 cm) and BUT had a negative one. Further analysis suggested that these correlations varied in different age groups. Multiple linear regression analysis indicated that main duct number (central 1 cm), rounding of lid margins, and hyperkeratinization significantly affected OSDI score.* Conclusions*. Close attention should be paid to the morphology and structure of the eyelid margin and the function of meibomian gland in the diagnosis, treatment, and follow-up of dry eye diseases.

## 1. Introduction

Dry eye is a multifactorial ocular surface disorder which is usually caused by chronic inflammation and characterized by tear film instability and increased osmolarity [1, 2]. There are various symptoms in dry eye patients, such as ocular discomfort, fluctuating visual disturbances, and potential damage [3]. These symptoms could cause impairment to the patients' quality of life [4].

Stern et al. [5] put forward the concept of lacrimal function unit (LFU), which consists of the main lacrimal gland, the ocular surface (cornea, conjunctiva, accessory lacrimal glands, and meibomian glands), and the interconnecting innervation. If any part of this functional unit is compromised, the ocular surface will be damaged [5]. The integrity of lacrimal functional unit plays a very important role in maintaining the stability of ocular surface [5, 6].

Dry eye disease is the most common ocular surface disorder. It affects up to 1/5 of the population and the prevalence increases with age [7]. Young perimenopausal and menopausal women are more vulnerable to this disease. In recent years, the prevalence of dry eye disease has been rising due to the aging of population and the increasing use of computer, air conditioner, and car.

The physical examinations of dry eye include Schirmer test, upper and lower tear meniscus height, tear film breakup time, corneal fluorescein staining, and construction and function of meibomian gland. However, research on changes of eyelid edge shape and the relationship between symptoms and signs of dry eye patients is quite rare. In this study, we screened out 19 ocular surface signs associated with dry eye symptoms through a thorough literature review [8, 9]. These signs include rounding of lid margins, notching of lid margins, vascularity of lid margins, lashes abnormity, hyperkeratinization, hyperemia of lid margins, main duct number (central 1 cm), plugging of orifices, scarred obliteration of orifices, main duct dropout, properties of the secretion, tear film breakup time (BUT), tear film fragment, tear film foam, upper tear meniscus height, lower tear meniscus height, conjunctival congestion, conjunctivochalasis, and corneal fluorescein staining. They can comprehensively reflect the ocular surface condition. The symptoms of dry eyes were evaluated with OSDI score. Through correlation analysis, we further studied the relationship between symptoms and signs of dry eye patients, providing a new insight in the diagnosis, treatment, and follow-up of dry eye disease.

## 2. Materials and Methods

### 2.1. Study Population

This is a prospective observational study. Altogether 176 subjects (176 eyes), including 60 males (60 eyes) and 116 females (116 eyes), were recruited from patients admitted to Peking University eye center affiliated to Peking University third hospital from June 1st to October 31st, 2014. Patients were divided into four age groups, with Group 1 under 30 years old (*N* = 45), Group 2 between 31 and 45 years old (*N* = 47), Group 3 between 46 and 60 years old (*N* = 44), and Group 4 over 60 years old (*N* = 40). Informed consent was obtained from all participants, and the study was approved by the Institutional Review Board of Peking University Third Hospital. Investigations were conducted in accordance with the tenets of the Declaration of Helsinki.

### 2.2. Inclusion and Exclusion Criteria

*Inclusion Criteria [3]*. ① eye symptoms (at least 1 item): dry, burning, mild itching, photophobia, and so on; ② a rapid tear breakup time; ③ Schirmer I test ≤10 mm/5 min; ④ ocular surface lesions (punctate staining with fluorescein dyes). The diagnosis of dry eye disease can be made when we have ① + ② (≤5 s) or ① + ② (≤10 s)+ ③, while ③ + ④ can enhance such diagnosis. If just a single eye meets the diagnosis standards, it will be selected; if both eyes meet diagnosis standards, the right eye will be selected.

*Exclusion Criteria*. Patients with recurrent inflammation or eye traumas were excluded. Patients who received eye operation within three months, wore contact lens within two weeks, or were with poor general condition such as poor blood sugar control, poor blood pressure control, and any other systemic disease that might affect the study were also excluded.

### 2.3. Research Methods

The symptoms of dry eye disease were assessed with the ocular surface disease index (OSDI), while the signs of each enrolled patient were measured by the same doctor in the following order: tear film, lid margin, meibomian glands, conjunctiva, and cornea. Slit-lamp microscope (TOPCON, PS-11E) was used for ocular surface examination. 

*Tear Film*. Tear film breakup time (BUT) test was used to assess the stability of tear film and was measured three times for each eye, from which an average value was calculated and adopted; tear fragment, which refers to the excess mucus fragments or debris in the tear film, was examined by distortion of the light reflexes on the cornea. It was graded on a dichotomous scale; tear foam in the tear film suggesting meibomian gland dysfunction was also graded on a dichotomous scale; the patients were required to look at the front horizontally, and the upper and lower tear meniscus height was measured and graded as 0 (<0.1 mm), 1 (0.1 mm to <0.2 mm), 2 (0.2 mm to <0.3 mm), or 3 (≥0.3 mm). 

*Conjunctiva [10]*. according to Institute for Eye Research (IER), conjunctival congestion was graded as 0 (no congestion), 1 (congestion was confined to the fornix and blood vessel was bright red), 2 (congestion was obvious and reached to palpebral fissure and blood vessel was crimson and fuzzy), or 3 (congestion was diffuse, blood vessel was fuchsia, and meibomian gland texture was not clear); according to LIPCOF (Lid-parallel conjunctival folds) [11, 12], conjunctivochalasis was graded as 0 (no obvious fold), 1 (single fold), 2 (2 folds, but the height of the fold was lower than the height of tear film), or 3 (many folds, and the height of the fold was higher than the height of tear film). 

*Cornea*. the cornea was divided into 4 quadrants. After fluorescein staining, each quadrant was scored separately: 0 (no staining), 1 (<5 points), 2 (≥5 points), or 3 (≥5 points and filaments or clumps staining); then the sum of the 4 quadrants (0–12 scores) was obtained and graded as 0 (0 score), 1 (1–4 scores), 2 (5–8 scores), and 3 (9–12 scores) [13]. 

*Lid Margins [8, 9]*. Six features were graded on a dichotomous scale, including rounding of lid margins, notching of lid margins, vascularity of lid margins, lashes abnormity, hyperkeratinization, and hyperemia of lid margins. 

*Meibomian Glands [8, 9, 14]*. Main duct number (central 1 cm) of upper eyelid was graded from 0 (no duct) to 5 (5 ducts); plugging of central 5 meibomian gland orifices of lower eyelid was graded as 0 (no orifice plugging), 1 (1-2 orifices plugging), 2 (3-4 orifices plugging), or 3 (all orifices plugging); scarred obliteration of central 10 meibomian gland orifices of upper eyelid was graded as 0 (no scarred obliteration), or 1 (loss of normal structure of meibomian gland orifices); main duct dropout of central 2/3 area of lower eyelid was graded as 0 (no dropout), 1 (less than 33% area dropout), 2 (34%–66% area dropout), or 3 (more than 67% area dropout); properties of the secretion were graded as 0 (clear secretion), 1 (mild muddy secretion), 2 (muddy, viscous, and granular secretion), or 3 (toothpaste-like secretion).

### 2.4. Statistical Analyses

Statistical analysis was performed using SPSS l3.0 (SPSS, Inc, Chicago, IL, USA). Partial correlation analysis was made to eliminate possible biases from age. After normal *W* test, Pearson correlation analysis was used for normally distributed data and Spearman correlation analysis was adopted for the abnormally distributed data. Correlation analysis was performed between OSDI score and the 19 ocular surface signs of patients in each age group. Correlation analysis among OSDI, the ocular signs and age was also performed. Multiple linear regression analysis was used to further analyze the extent of the influences by these signs, in which OSDI score and the related signs were, respectively, dependent variable and independent variables. All the statistical tests are two-tailed tests.

## 3. Results

### 3.1. Demographics

There were totally 176 patients (176 eyes), including 60 males (60 eyes) and 116 females (116 eyes) in this study. The average age of the patients was 45.84 ± 17.46 years old (ranging from 7 to 86 years); the average OSDI score was 28.30 ± 9.752 (ranging from 12.5 to 59). Among the 176 patients, the distributions of ocular surface symptoms and signs are listed in Tables 1, 2, and 3.

### 3.2. Correlation Analysis between OSDI Score and Signs

**Figure 1:**
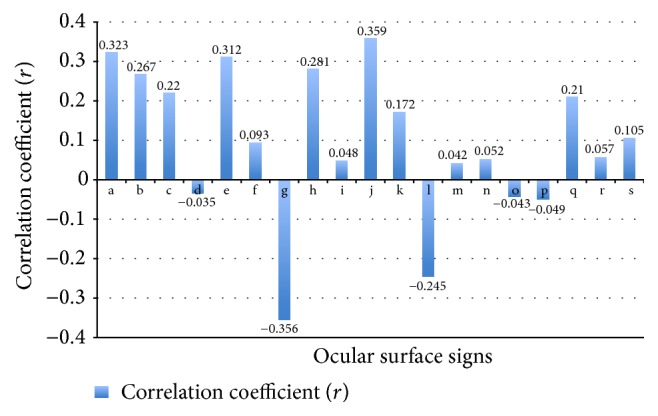
Correlation coefficient (*r*) between ocular surface signs and OSDI score.* Note*. Abscissa axis: a: rounding of lid margins, b: notching of lid margins, c: vascularity of lid margins, d: lashes abnormity, e: hyperkeratinization, f: hyperemia of lid margins, g: main duct number (central 1 cm), h: plugging of orifices, i: scarred obliteration of orifices, j: main duct dropout, k: properties of the secretion, l; BUT, m: tear film fragment, n: tear film foam, o: upper tear meniscus height, p: lower tear meniscus height, q: conjunctival congestion, r: conjunctivochalasis, and s: corneal fluorescein staining; vertical axis: correlation coefficient *r*.

Partial correlation analysis was performed between OSDI score and the 19 ocular surface signs, with the age as a control variable (Table 4). Signs including rounding of lid margins, notching of lid margins, vascularity of lid margins, hyperkeratinization, plugging of orifices, main duct dropout, and conjunctival congestion showed a positive correlation with OSDI score (*P* < 0.005), while main duct number (central 1 cm) and BUT showed a negative correlation with OSDI score (*P* < 0.001). The correlation coefficient *r* ranged from −0.356 to 0.359 (Figure 1). The remaining signs did not show any statistically significant correlations with OSDI score.

Correlation analysis by age groups was also performed between OSDI score and the 19 ocular surface signs (Table 5). Signs that were statistically correlated to OSDI also showed a different correlation coefficient in different age groups. Further correlation analysis between age and signs was also performed (Table 6). Rounding of lid margins, vascularity of lid margins, lashes abnormity, hyperkeratinization, hyperemia of lid margins, plugging of orifices, scarred obliteration of orifices, upper tear meniscus height, conjunctival congestion, and corneal fluorescein staining were found to be correlated with age (*P* < 0.001). The correlation analysis between age and OSDI was also statistically significant (*r* = 0.451, sig = 0.000).

### 3.3. Multiple Linear Regression Analysis between OSDI Score and Signs

Through correlation analysis, twelve signs were found correlated with OSDI score. In order to further determine which signs have the greatest impact on OSDI score, multiple linear regression was performed, with OSDI score as dependent variable and the related signs as independent variables (Table 7). The regression equation was OSDI = 28.08 + main duct number (central 1 cm) × (−0.285) + rounding of lid margins × 0.229 + hyperkeratinization × 0.201; the results showed that main duct number (central 1 cm), rounding of lid margins, and hyperkeratinization had the most significant influences on OSDI score.

## 4. Discussion

The integrity of lacrimal functional unit plays a very important role in maintaining the stability of ocular surface [5, 6]. If any part of this functional unit is compromised, the ocular surface will be damaged [5]. The occurrence of dry eye is mainly associated with high osmolarity of tears, which may activate a series of inflammatory reactions and lead to disorders of morphology, structure, and function on the long run. From this perspective, the ocular surface signs related to morphology, structure, and function were studied.

To find out the correlations between dry eye symptoms and signs, we screened out 19 ocular surface signs associated with dry eye symptoms based on a thorough literature review [8, 9], as mentioned in the introduction. We used OSDI questionnaire to measure the symptoms of patients. OSDI is an internationally recognized index to evaluate the severity of ocular surface disease. The higher the score is, the worse the ocular surface condition. It is considered to be objective, efficient, and accurate and consists of three parts: eye discomfort, visual function, and tolerance to environmental factors [15].

Given the clear relation between dry eye disease and age. Partial correlation taking age as a control variable was performed. Signs including rounding of lid margins, notching of lid margins, vascularity of lid margins, hyperkeratinization, plugging of orifices, main duct dropout, and conjunctival congestion showed a positive correlation with OSDI score (*P* < 0.005), while main duct number (central 1 cm) and BUT showed a negative correlation with OSDI score (*P* < 0.001). Signs that were statistically correlated to OSDI also showed different correlation coefficients among different age groups. This complexity could be explained in three ways. First of all, the sample of this study consists of 176 eyes, which were divided into 4 groups. The subject number in the groups ranges from 40 to 47, which may not be ideal for age group study. Secondly, we used Pearson and Spearman analysis for correlation study, which only tested linear correlation. There might be other kind of correlations between these signs and OSDI, so the statistics might cause confusion when the correlation coefficient showed irregular tendency. Thirdly, age might not affect the correlation between signs and symptoms; the difference among different groups might be caused by the respective correlation between signs or OSDI and age. For that case, further correlation analysis between age and signs as well as with OSDI was, respectively, performed. Rounding of lid margins, vascularity of lid margins, lashes abnormity, hyperkeratinization, hyperemia of lid margins, plugging of orifices, scarred obliteration of orifices, upper tear meniscus height, conjunctival congestion, and corneal fluorescein staining were found to be correlated with age (*P* < 0.001). This correlation accords with the fact that dry eyes are more severe in order people, which may result in the difference of the correlation between signs and OSDI among different age groups. But as the correlation coefficient is low to moderate, further research with larger samples is needed to provide more convincing explanation.

### 4.1. Correlation Analysis between OSDI Score and Tear Film, Conjunctiva, and Cornea Signs

Taking age as a control variable, results from partial correlation analysis showed that conjunctival congestion (*r* = 0.210, *P* = 0.005) and conjunctivochalasis (*r* = 0.057, *P* = 0.455) were positively correlated to OSDI score, while BUT (*r* = −0.245, *P* = 0.000) was negatively correlated to OSDI score. This indicates that the more serious the conjunctival congestion and conjunctivochalasis and the lower the BUT, the worse the symptoms of dry eye. Signs that were not statistically correlated to OSDI score were tear film fragment (*r* = 0.042, *P* = 0.583), tear film foam (*r* = 0.052, *P* = 0.494), upper tear meniscus height (*r* = −0.043, *P* = 0.572), lower tear meniscus height (*r* = −0.049, *P* = 0.523), and corneal fluorescein staining (*r* = 0.105, *P* = 0.167). Further study is needed to explore the interrelations between these signs. A possible reason for these results may be that when MGD occurred, the changes of lipid composition and quantity would affect the stability of tear film, causing the decline of BUT. Research [11] showed that there was significant correlation between conjunctivochalasis and MGD, that is, the heavier the degree of conjunctivochalasis, the higher the incidence of MGD. As for the negative results on tear film fragment, tear film foam, upper and lower tear meniscus height, and corneal fluorescein staining, previous studies indicate that there might be some correlation between these signs and the severity of dry eye symptoms [16]. However, these signs are also affected by the interference factors of the existing dry eye examination method and the disadvantages of the repeatability of the method. The poor correlations between these signs and symptoms suggest that they are not suitable for the evaluation of dry eye diseases.

### 4.2. Correlation Analysis between OSDI Score and Lid Margins Signs

The results of this study indicated that the morphology and structure of the ocular surface and the eyelid were very significantly correlated with OSDI score. The top four signs that were correlated with OSDI were rounding of lid margins (*r* = 0.323, *P* = 0.000), hyperkeratinization (*r* = 0.312, *P* ≤ 0.000), notching of lid margins (*r* = 0.627, *P* = 0.001), and vascularity of lid margins (*r* = 0.22, *P* = 0.000). We do not know if there was any interrelations among some of the ocular characteristics and we could not avoid collinearity. But this correlation indicated that we should pay more attention to the morphology and structure of the ocular surface and the eyelid in the diagnosis, treatment, and follow-up. The possible causal relationships between these signs and symptoms could be further studied.

Bron et al. [17, 18] found that there is a certain osmotic pressure gradient of tear film. Osmolarity is relatively higher at the top of tear film and eyelid edge. The concentration of inflammatory factors and other harmful substances are the highest near lid margins. Palpebral margin cells have a higher permeability of these harmful substances, which triggers the series of pathological and physiological process, resulting in pathological damage of the palpebral margin (such as eyelid keratosis). Lemp et al. [19] showed that hyperosmolarity is related to dry eye diseases with high sensitivity and specificity. It indicates that abnormal signs of palpebral margin may exist throughout the process of occurrence and development of dry eye. Our study further echoed this theory, suggesting more attention on lid margins abnormal changes of dry eye patients.

### 4.3. Correlation Analysis between OSDI Score and Meibomian Gland Signs

As indicated in this study, OSDI score was positively associated with the score of main duct dropout (*r* = 0.359, *P* = 0.000), plugging of orifices (*r* = 0.281, *P* = 0.000), and properties of the secretion (*r* = 0.172, *P* = 0.000), while negatively with main duct number (central 1 cm) (*r* = −0.356, *P* = 0.000). In other words, the more serious the plugging of orifices, main duct dropout, and properties of the secretion and the lower the main duct number (central 1 cm), the heavier the symptoms of dry eye. It also indicates that corresponding treatment regarding these signs can be carried out to relieve the symptoms of dry eye.

It is reported that about 78% of dry eyes are caused by MGD, and the prevalence of MGD was estimated to be 46.2%–69.3% in people over 40 years in Asia [20, 21]. The average age of the patients in this study was 45.84 years, and 91.5% of the dry eye subjects in this study were also MGD patients. Bron et al. [17, 18] reported that pathogenesis of MGD might be associated with eyelid margin lesions caused by elevating of tear osmolarity. It indicates that palpebral margin disorder is closely related to the function of the meibomian gland. Researches [9, 17, 18, 21, 22] show that osmolarity of tear film is relatively higher near lid margins, where harmful substances will increase as well. This may lead to damage, apoptosis, and even failure of ocular surface epithelial cells and lid margins stem cells. If the damage is faster than the recovery of stem cells, palpebral margin disorder will occur, which may trigger or accelerate the process of duct keratinization around meibomian gland orifices or plugging of orifices, eventually leading to MGD. MGD would increase the osmolarity of tear film as well. To sum up, this is a vicious cycle of pathophysiological processes.

### 4.4. Multiple Linear Regression Analysis between OSDI Score and Signs

According to the regression equation, main duct number (central 1 cm), rounding of lid margins, and hyperkeratinization were the top three factors that had the most significant influences on OSDI score. Though we do not know the cause-and-effect relationship between the signs and symptoms, it suggests that we should pay more attention to the morphology and structure in the diagnosis, treatment, and follow-up of meibomian gland disease and eyelid margin disease.

## 5. Conclusion

This study identifies 12 signs which are related to OSDI score, including rounding of lid margins, notching of lid margins, vascularity of lid margins, hyperkeratinization, hyperemia of lid margins, main duct number (central 1 cm), plugging of orifices, main duct dropout, properties of the secretion, tear film breakup time (BUT), conjunctival congestion, and conjunctivochalasis. Main duct number (central 1 cm), rounding of lid margins, and hyperkeratinization are the most significant factors that influence OSDI score. More attention should be paid to the morphology and structure of the eyelid margin and the function of meibomian gland in the diagnosis, treatment, and follow-up of dry eye diseases.

## Figures and Tables

**Table 1:** Ocular surface signs of dye eye patients.

Signs	Absent number (%)	Present number (%)
Rounding of lid margins	103 (58.5)	73 (41.5)
Notching of lid margins	111 (63.1)	65 (36.9)
Vascularity of lid margins	107 (60.8)	69 (39.2)
Lashes abnormity	174 (98.9)	2 (1.1)
Hyperkeratinization	44 (25.0)	132 (75.0)
Hyperemia of lid margins	16 (9.1)	160 (90.9)
Scarred obliteration of orifices	171 (97.2)	5 (2.8)
Tear film fragment	15 (8.5)	161 (91.5)
Tear film foam	146 (83.0)	30 (17.0)

**Table 2:** Ocular surface signs of dye eye patients.

Signs	0 number (%)	1 number (%)	2 number (%)	3 number (%)
Plugging of orifices	15 (8.5)	51 (29.0)	73 (41.5)	37 (21.0)
Main duct dropout	31 (17.6)	62 (35.2)	60 (34.1)	23 (13.1)
Properties of the secretion	11 (6.3)	43 (24.4)	88 (50.0)	34 (19.3)
Upper tear meniscus height	51 (29.0)	77 (43.8)	44 (25.0)	4 (2.3)
Lower tear meniscus height	37 (21.0)	77 (43.8)	53 (30.1)	9 (5.1)
Conjunctival congestion	19 (10.8)	124 (70.5)	32 (18.1)	1 (0.6)
Conjunctivochalasis	144 (81.8)	27 (15.3)	3 (1.7)	2 (1.2)
Corneal fluorescein staining	0 (0.0)	166 (94.9)	4 (2.3)	5 (2.8)

*Note*. The grading of each sign is stated in the method part.

**Table 3:** Ocular surface symptom and signs of dye eye patients.

	Mean	SD	*N*
OSDI	29.3920	9.64497	176
Main duct number (central 1 cm)	5.3466	3.58398	176
BUT	2.2841	1.25992	176

**Table 4:** Partial correlation analysis between OSDI scores and signs.

Control		a	b	c	d	e	f	g	h	i	j	k	l	m	n	o	p	q	r	s
Age	*r*	.323	.267	.220	−.035	.312	.093	−.356	.281	.048	.359	.172	−.245	.042	.052	−.043	−.049	.210	.057	.105
	*P* value	.000	.000	.003	.644	.000	.220	.000	.000	.529	.000	.023	.001	.583	.494	.572	.523	.005	.455	.167

*Note*. a: rounding of lid margins, b: notching of lid margins, c: vascularity of lid margins, d: lashes abnormity, e: hyperkeratinization, f: hyperemia of lid margins, g: main duct number (central 1 cm), h: plugging of orifices, i: scarred obliteration of orifices, j: main duct dropout, k: properties of the secretion, l: BUT, m: tear film fragment, n: tear film foam, o: upper tear meniscus height, p: lower tear meniscus height, q: conjunctival congestion, r: conjunctivochalasis, s:corneal fluorescein staining, and *r*: correlation coefficient.

**Table 5:** Correlation analysis between OSDI score and signs.

Signs	Overall		Age Group 1		Age Group 2		Age Group 3		Age Group 4	
	*r*	*P* value	*r*	*P* value	*r*	*P* value	*r*	*P* value	*r*	*P* value
Rounding of lid margins	0.504	0.000^*∗∗∗*^	0.042	0.785	0.445	0.002^*∗∗*^	0.595	0.000^*∗∗∗*^	0.355	0.025^*∗*^
Notching of lid margins	0.388	0.000^*∗∗∗*^	0.077	0.617	0.365	0.012	0.502	0.000^*∗∗∗*^	0.246	0.125
Vascularity of lid margins	0.31	0.000^*∗∗∗*^	−0.011	0.944	0.173	0.242	0.335	0.026	0.33	0.037
Lashes abnormity	−0.084	0.266	—	—	−0.161	0.279	—	—	—	—
Hyperkeratinization	0.429	0.000^*∗∗∗*^	0.301	0.044	0.375	0.009^*∗∗*^	0.539	0.000^*∗∗∗*^	0.265	0.099
Hyperemia of lid margins	0.21	0.005	0.188	0.215	0.085	0.568	0.181	0.239	—	—
Main duct number (central 1 cm)	−0.526	0.000^*∗∗∗*^	−0.326	0.029	−0.32	0.028	−0.549	0.000^*∗∗∗*^	−0.507	0.000^*∗∗∗*^
Plugging of orifices	0.379	0.000^*∗∗∗*^	0.251	0.096	0.203	0.171	0.371	0.013^*∗*^	0.359	0.023^*∗*^
Scarred obliteration of orifices	0.134	0.077	—	—	—	—	0.242	0.114	−0.029	0.859
Main duct dropout	0.485	0.000^*∗∗∗*^	0.257	0.089	0.403	0.005^*∗∗*^	0.483	0.000^*∗∗∗*^	0.529	0.000^*∗∗∗*^
Properties of the secretion	0.258	0.000^*∗∗∗*^	−0.065	0.673	0.245	0.097	0.392	0.009	0.043	0.793
BUT	−0.252	0.000^*∗∗∗*^	−0.039	0.799	−0.384	0.008^*∗∗*^	−0.233	0.128	−0.328	0.039^*∗*^
Tear film fragment	0.091	0.229	0.339	0.023^*∗*^	−0.003	0.984	−0.103	0.507	−0.195	0.228
Tear film foam	0.113	0.135	0.187	0.217	−0.194	0.191	−0.065	0.674	−0.066	0.687
Upper tear meniscus height	−0.064	0.398	0.001	0.995	0.088	0.559	−0.122	0.429	−0.25	0.12
Lower tear meniscus height	−0.056	0.459	−0.048	0.752	0.055	0.713	−0.089	0.566	−0.134	0.411
Conjunctival congestion	0.209	0.005^*∗∗*^	0.081	0.595	0.202	0.174	0.386	0.010^*∗∗*^	0.165	0.309
Conjunctivochalasis	0.218	0.004^*∗∗*^	−0.012	0.939	0.37	0.010^*∗∗*^	−0.037	0.811	0.04	0.808
Corneal fluorescein staining	0.097	0.201	0.227	0.134	0.084	0.576	0.012	0.938	0.08	0.624

Note: ^*∗∗∗*^*P* < 0.001; ^*∗∗*^*P* < 0.01; ^*∗*^*P* < 0.05.

**Table 6:** Correlation analysis between signs and age.

	a	b	c	d	e	f	g	h	i	j	k	l	m	n	o	p	q	r	s
*r*	.285^*∗∗*^	−.075	.375^*∗∗*^	.328^*∗∗*^	−.599^*∗∗*^	.427^*∗∗*^	.191^*∗*^	.537^*∗∗*^	.280^*∗∗*^	.139	.134	−.071	−.077	.052	.436^*∗∗*^	−.059	.285^*∗∗*^	−.075	.375^*∗∗*^
*P* value	.000	.323	.000	.000	.000	.000	.011	.000	.000	.066	.077	.350	.312	.496	.000	.439	.000	.323	.000

*Note*. a: rounding of lid margins, b: notching of lid margins, c: vascularity of lid margins, d; lashes abnormity, e: hyperkeratinization, f: hyperemia of lid margins, g: main duct number (central 1 cm), h: plugging of orifices, i; scarred obliteration of orifices, j: main duct dropout, k: properties of the secretion, l; BUT, m; tear film fragment, n; tear film foam, o: upper tear meniscus height, p; lower tear meniscus height, q: conjunctival congestion, r: conjunctivochalasis, s: corneal fluorescein staining, and *r*: correlation coefficient.

**Table 7:** Multiple linear regression analysis between OSDI score and signs.

Sign	Regression coefficient B	*P* value
Rounding of lid margins	0.506	<0.001
Notching of lid margins	0.396	<0.001
Vascularity of lid margins	0.325	<0.001
Hyperkeratinization	0.446	<0.001
Hyperemia of lid margins	0.264	<0.001
Main duct number (central 1 cm)	−0.541	<0.001
Plugging of orifices	0.449	<0.001
Main duct dropout	0.534	<0.001
Properties of the secretion	0.310	<0.001
BUT	−0.290	<0.001
Conjunctival congestion	0.209	0.003
Conjunctivochalasis	0.240	0.001
